# Optimization of differential filtration-based mitochondrial isolation for mitochondrial transplant to cerebral organoids

**DOI:** 10.1186/s13287-023-03436-y

**Published:** 2023-08-15

**Authors:** David F. Bodenstein, Pavel Powlowski, Kassandra A. Zachos, Dana El Soufi El Sabbagh, Hyunjin Jeong, Liliana Attisano, Landon Edgar, Douglas C. Wallace, Ana Cristina Andreazza

**Affiliations:** 1 Department of Pharmacology and Toxicology, University of Toronto, Medical Science Building, Room 4211, 1 King’s College Circle, Toronto, ON M5S 1A8 Canada; 2 Department of Biochemistry, University of Toronto, Toronto, ON Canada; 3 Donnelly Centre, University of Toronto, Toronto, ON Canada; 4 Department of Chemistry, University of Toronto, Toronto, ON Canada; 5 Department of Biological Chemistry, Center for Molecular and Mitochondrial Medicine and Genetics, University of California, Irvine, CA USA; 6 Division of Human Genetics, Department of Pediatrics, Center for Mitochondrial and Epigenomic Medicine, Children’s Hospital of Philadelphia, Philadelphia, USA; 7 Department of Pediatrics, The Perelman School of Medicine, University of Pennsylvania, Philadelphia, PA USA; 8 Department of Psychiatry, University of Toronto, Toronto, ON Canada

**Keywords:** Mitochondria, Mitochondrial isolation, Mitochondrial transplant, Induced pluripotent stem cells, Cerebral organoids

## Abstract

**Background:**

Mitochondrial dysfunction is involved in several diseases ranging from genetic mitochondrial disorders to chronic metabolic diseases. An emerging approach to potentially treat mitochondrial dysfunction is the transplantation of autologous live mitochondria to promote cell regeneration. We tested the differential filtration-based mitochondrial isolation protocol established by the McCully laboratory for use in cellular models but found whole cell contaminants in the mitochondrial isolate.

**Methods:**

Therefore, we explored alternative types of 5-μm filters (filters A and B) for isolation of mitochondria from multiple cell lines including HEK293 cells and induced pluripotent stem cells (iPSCs). MitoTracker™ staining combined with flow cytometry was used to quantify the concentration of viable mitochondria. A proof-of-principle mitochondrial transplant was performed using mitoDsRed2-tagged mitochondria into a H9-derived cerebral organoid.

**Results:**

We found that filter B provided the highest quality mitochondria as compared to the 5-μm filter used in the original protocol. Using this method, mitochondria were also successfully isolated from induced pluripotent stem cells. To test for viability, mitoDsRed2-tagged mitochondria were isolated and transplanted into H9-derived cerebral organoids and observed that mitochondria were engulfed as indicated by immunofluorescent co-localization of TOMM20 and MAP2.

**Conclusions:**

Thus, use of filter B in a differential filtration approach is ideal for isolating pure and viable mitochondria from cells, allowing us to begin evaluating long-term integration and safety of mitochondrial transplant using cellular sources.

**Supplementary Information:**

The online version contains supplementary material available at 10.1186/s13287-023-03436-y.

## Background

Mitochondria are found in almost every body cell type and are responsible for the regulation of chemical energy production (adenosine triphosphate, ATP), calcium buffering, heat production, cellular death, and others [1]. As expected, mitochondrial dysfunction can cause a wide range of clinical features and diseases [1]. Evidence of mitochondrial dysfunction has been reported in several chronic diseases such as diabetes, cardiovascular disease, Parkinson’s and Alzheimer’s disease, bipolar disorder, and schizophrenia [2–6]. Mitochondrial dysfunction can be caused by an impairment in oxidative phosphorylation (OXPHOS), mitochondrial fission/fusion dynamics, or mitochondrial and nuclear DNA (mtDNA; nDNA) mutations/deletions [3, 7–9]. In severe cases, patients with mitochondrial dysfunction can be diagnosed with rare mitochondrial diseases, such as Leber’s Hereditary Optic Neuropathy (LHON) [8], Myoclonic Epilepsy with Ragged Red Fibers (MERRF) [10], and mitochondrial myopathy, encephalopathy, lactic acidosis, and stroke-like episodes (MELAS) [11], which are all caused by mutations in the mtDNA.

Obtaining a definitive diagnosis for a mitochondrial disease through histological/biochemical analysis and whole genome sequencing can provide guidance for treatment options based on the disease prognosis [1]. However, reaching a definitive diagnosis is complicated by the heterogeneity of clinical features and variation in age of onset [1]. The use of animal and cellular models has advanced the understanding of mitochondrial disease pathology to assist in diagnosis. The development of mouse models for LHON with the same mt-ND6 amino acid substitution as found in patients has provided insight into novel therapeutic options and understanding of the LHON pathology [12]. Similarly, the generation of cybrid cell models has allowed researchers to create cells with known mitochondrial and nuclear backgrounds to identify pathogenic mutations and the etiology of mitochondria dysfunction [13]. The isolation of mitochondria has been critical to evaluate biochemical and functional properties, such as OXPHOS complex activity and protein trafficking/import to mitochondria [14, 15]. By combining these models, Emani et al. [16] evaluated the use of autologous mitochondrial transplants to restore mitochondrial function following ischemia–reperfusion injury in the myocardium. By adopting this method for mitochondrial disease, it presents a novel therapeutic approach to transplant wildtype mtDNA into tissues with high levels of mutant mtDNA. Interestingly, Shoffner et al. and Chomyn et al*.* found that increasing wildtype mtDNA by a small percentage had protective effects in MERRF and MELAS, respectively [10, 17].

Mitochondria can be isolated from tissue and cells using several methods for biochemical analysis and transplant. The most common method is differential centrifugation, which relies on low- and high-speed centrifugation steps to pellet mitochondria [18]. Overall, this method is easily performed with minimal specialized equipment; however, it relies on repetitive centrifugation steps, which increase the isolation time and reduce mitochondrial viability [19]. To mitigate this issue, Preble et al., developed a filtration-based mitochondrial isolation method for muscle tissue, which reduces isolation time to 30 min and has been evaluated clinically in pediatric patients with congenital heart disease [16, 19]. We tested this protocol for use in cells but found whole cells contamination in the mitochondrial isolate. Therefore, we evaluated the use of new 5-μm filters with unique membrane compositions compared to the original 5-μm pluriselect filter used by Preble et al. (pluriselect: PET membrane; filter A: nylon membrane; filter B: PVDF membrane) [19]. Mitochondria were isolated from cell lines and induced pluripotent stem cells (iPSCs) with each filter and assessed for mitochondrial quality and purity. By evaluating mitochondrial quality with flow cytometry, qPCR, TEM, and mitochondrial transplants in H9 stem-cell derived cerebral organoids (COs), we identified filter B to be optimal for isolating mitochondria from diverse cellular models.

## Methods

### Cell culture

Cell lines (HEK293, MDA-MB-231, and MCF7) were cultured following the ATCC guidelines. Specifically, cells were maintained in T25, T75, and T175 culture flasks in DMEM (Gibco, #11995065) with 10% FBS (Wisent, #98150) and 1% penicillin/streptomycin (Gibco, #15140122). Osteosarcoma 143B(TK-) ρ0 cells were obtained from Dr. Douglas Wallace’s group and were also maintained in DMEM containing FBS and penicillin/streptomycin with additional 50 μg/ml uridine supplementation [20]. HEK293 cells were transfected with mitoDsRed2 using lipofectamine LTX (Invitrogen, #15338100) following the manufacturer’s methods. DsRed2-Mito-7 (mitoDsRed2) was a gift from Michael Davidson (Addgene plasmid #55838; http://n2t.net/addgene:55838; RRID: Addgene_55838). Cells were used within a week after transfection.

Induced pluripotent stem cells were generated from commercially purchased (American Type Culture Collection [ATCC]) human peripheral blood mononuclear cells (PBMC) using previously described methods [21, 22]. iPSCs were maintained on 1% geltrex coated 6-well plates and cultured in mTeSR™ plus basal medium (Stem Cell Technologies, #100-0276), and characterized by examining alkaline phosphatase, pluripotency marker expression, and normal karyotype, as described by Marti et al. [23]. The detailed method for each characterization is published in Duong et al. [21]. Briefly, iPSCs were stained with the Alkaline Phosphatase (AP) Live Stain and Pluripotent Stem Cell 4-Marker Immunocytochemistry Kit (Invitrogen, A14353 and A24881, respectively) to detect key pluripotency markers. mRNA expression of pluripotency markers were further validated using pre-designed RT^2^ qPCR primers on *SOX2*, *POUF5F1*, *KLF4*, *MYCL1*, *LIN28A* and normalized to *ACTB* and *GAPDH*. Lastly, karyotype was analyzed by G-banding performed by TCAG Cytogenomics Facility (The Hospital for Sick Children, Toronto, ON, Canada).

H9 embryonic stem cells were grown in 6 well tissue culture plates using Mouse embryonic fibroblast conditioning medium (MEF-CM) and once cells reached 70–80% confluency, colonies were split using TrypLE dissociation reagent. Cells were singularized and seeded at 12,000 cells/well in a 96 well V-bottom plate to induce embryoid body (EB) formation. On day 4, EBs were scanned and picked to transfer to an ultra-low attachment 24 well plate for neuroepithelium induction of EBs. On day 7, EBs were embedded in Matrigel droplets to allow neural bud formation and expansion. On days 11 or 12, COs were cut out of the Matrigel and placed in non-tissue culture 6 well plates on the shaker with organoid maturation medium as previously reported [21, 22, 24]. A media change was done twice a week with 3 ml of media per well to mature the COs until ready for use at 5 months.

### Mitochondrial isolation

The protocol to isolate mitochondria was modified from a previously published method from Preble et al*.* [19]. Mitochondria were isolated from 4 cell types, specifically HEK293, MDA-MB-231, MCF7, and iPSC. Cells were typically cultured in T175 culture flasks, to yield 2.5–3.5 × 10^7^ cells/flask to allow for parallel mitochondrial isolations using ~ 10^7^ cells each. Once cells were collected and pelleted, cells were resuspended in 2 ml of syringe-filtered (0.22-μm Acrodisc^®^ Syringe filter, PALL Life Sciences & 0.20-μm Filtropur Syringe Filter, Sarstedt) homogenization buffer (300 mM sucrose, 10 mM K-HEPES [pH 7.2], and 1 mM K-EGTA [pH 8.0]) and homogenized in gentleMACS C tubes using the gentleMACS Dissociator’s (Miltenyi Biotec, Bergisch Gladbach, North Rhine-Westphalia, Germany) pre-programmed mitochondrial setting. After homogenization, sample volume was completed to 5 ml/isolation. One milligram of Subtilisin A (Sigma-Aldrich, #P5380) was added to each sample and mixed by inversion and then incubated on ice for 10 min followed by centrifugation at 400* g* × 8 min at 4 °C. After centrifugation, the supernatant from each sample was pipetted 1 ml at a time through sterile and pre-washed 40-, 10-, and 5-μm filters (40 μm: Falcon, #352340, nylon membrane; 10 μm: Pluriselect, #43-50010-01, PET membrane; 5 μm: Pluriselect, #43-50005-13, PET membrane; 5 μm: Filter A, nylon membrane; 5 μm: Filter B, PVDF membrane). Filtrate was then collected in 1.5-ml microcentrifuge tubes and centrifuged at 9000* g* × 5 min at 4 °C. Pellets were resuspended and combined in 1.5 ml syringe-filtered respiration buffer (250 mM sucrose, 2 mM KH_2_PO_4_, 10 mM MgCl_2_, 20 mM K-HEPES [pH 7.2] and 0.5 mM K-EGTA [pH 8.0]) and washed three times. Mitochondria isolate was then divided into the relevant quality control experiments, specifically transmission electron microscopy (TEM), mtDNA content, and flow cytometry.

### Mitochondrial transplant

Mitochondria were transplanted into osteosarcoma 143B(TK-) ρ0 host cells and COs by co-incubation at 37 °C for 2 h. Mitochondrial transplant was based on previously published protocols [13, 25, 26]. Each protocol utilizes different aspects in their relative methods. The protocol that we have created bases on previously published protocols with adaptions. Briefly, for transplant into osteosarcoma 143B(TK-) ρ0 cells, mitochondria were isolated from HEK293 cells under sterile conditions in a biological safety cabinet. The final mitochondrial isolate was split 3 ways and subjected to flow cytometry and qPCR, for quality control, and for transplant. Isolated mitochondria are washed, protein concentration was determined by measuring absorbance at A280 and 12.5 μg of mitochondrial protein was added to the host cells and co-incubated at 37 °C for 2 h. After 2 h, media containing mitochondria was removed and replaced with fresh media. This time point was selected, as prior work has demonstrated that mitochondria are engulfed by host cells within 2 h [26–28], and due to the decline in the oxygen consumption rate of isolated mitochondria after 2 h [29]. After 24 h post-transplant, cell culture media was changed to remove uridine and pyruvate supplementation from 143B(TK-) cells to begin selection for cells that uptake viable mitochondria (DMEM, high glucose, L-glutamine, no sodium pyruvate, Gibco, #11965092).

For the mitochondrial transplant into H9-derived COs, mitochondria were isolated from HEK293 cells with DsRed2-tagged mitochondria (HEK293-mitoDsRed2), which allows tracking of penetrance and integration of donor mitochondria into host cells by fluorescent imaging. COs (4-month-old) were sliced at 400 μm thickness using a vibratome and cultured in a 96-well tissue culture plate with 150uL CO media for 24 h pre-transplant to allow the CO slices to recover after slicing. Mitochondria were transplanted at a volume of 133 μl (representing ~ 3.7 × 10^6^ isolated mitochondria) by adding the isolated mitochondria resuspended in CO media into the 96 well plate. Mitochondria were co-incubated with the CO for 2 h at 37 °C. After 2 h, cell culture media was removed and replaced with fresh media. While mitochondria were co-incubated with the CO slice, we determined the mitochondrial concentration by flow cytometry. This approach allowed us to initiate the mitochondrial transplant and then calculate the number of mitochondria transplanted based on the concentration.

### Confocal microscopy

Confocal microscopy was performed as previously described by Duong et al*.* [21]. Briefly, at 24 h post-mitochondrial transplant, CO were fixed with 4% PFA overnight at 4 °C, then washed and cryoprotected with 30% sucrose. The CO was embedded and cryosectioned into 20-μm-thick slices using a Leica cryostat and stored at − 80 °C until used. When samples were ready for use, the CO slices were washed with PBS-T (PBS pH 7.4, 0.1% Tween-20), then incubated for 1 h at room temperature in permeabilization/blocking solution (PBS-T pH 7.4, 0.5% TritonX-100, 2% BSA). Primary antibodies (TOMM20, 1:100, rabbit, abcam, ab186734; MAP2, 1:1000, chicken, abcam, ab5392), were prepared in antibody blocking solution (PBS-T pH 7.4, 0.5% BSA) and added to the tissue slices in a humidified chamber overnight at 4 °C. Samples were then washed with PBS-T and incubated for 1 h at room temperature with the appropriate secondary antibody (Donkey anti-Rabbit IgG [H + L] Highly Cross-Adsorbed Secondary Antibody Alexa Fluor Plus 647, Invitrogen, #A32795; Donkey anti-Chicken IgY [IgG] [H + L] Alexa Fluor^®^ 488, Jackson ImmunoResearch Laboratories Inc., #703-546-155) at 1:750 in antibody blocking solution. Coverslips were mounted with ProLong Gold Antifade Mountant with DAPI (Invitrogen, P36935). Samples were imaged using the confocal microscope (LSM 880 Elyra Superresolution) in the Microscopy Imaging Laboratory (MIL, University of Toronto, Canada).

### Transmission electron microscopy

Mitochondrial isolates were evaluated for purity and structure using transmission electron microscopy (TEM) in the U of T MIL. Mitochondrial isolates were fixed with a universal primary fixation buffer (4% paraformaldehyde and 1% glutaraldehyde in 0.1 M phosphate buffer) for 1 h at room temperature, then replaced with fresh fixation buffer for an overnight incubation in the fridge. Samples were then delivered to the MIL for secondary fixation, dehydration, polymerization, staining, and sectioning. Briefly, after primary fixation, samples were post-fixed in 1% osmium tetroxide in phosphate buffer and dehydrated with a graded series of ethanol and distilled water mixtures. Following dehydration, samples were washed with transitional solvent, propylene oxide, and infiltrated with a graded series of epoxy resin and propylene oxide mixtures. Polymerized samples were then sliced at 90 nm thickness using a Reichert Ultracut E microtome and collected on 300 mesh copper grids. Sections were then stained with saturated uranyl acetate, rinsed with distilled water, then Reynold’s lead citrate, and washed again with distilled water. Sections were visualized using a FEI Talos L120C transmission electron microscope at an accelerating voltage of 120 kV.

TEM images of isolated mitochondria were quantified using a semi-automated approach. We created a model using the Trainable Weka Segmentation [30] plug-in for Fiji [31] to classify objects as mitochondria, cellular debris, or background. Once mitochondria were identified by the model, we reviewed the images and refined the selections to remove mislabeled debris and grouped mitochondria. Additionally, engulfed and/or damaged mitochondria were excluded from the total mitochondrial count. Images were validated once more by a blinded individual to confirm the selection and remove any observer bias.

### mtDNA content

DNA was isolated to compare the relative quantities of mtDNA and nDNA in the whole cell compared to the mitochondrial isolate. Quantitative PCR (qPCR) was performed using MT-ND1 (mtDNA) and β2-microglobulin (nDNA) primers as described by Bodenstein et al*.* [32]. Briefly, DNA was isolated from 2 to 5 × 10^5^ cells or 100 μl of mitochondrial isolate using a proteinase K digestion followed by isopropanol precipitation. Following extraction of DNA, samples were diluted to 1 ng/μl and plated with Bioline 2X SensiFAST SYBR No-ROX (Catalog No. #BIO-98050). qPCR was performed on the Bio-Rad CFX96 (Bio-Rad Laboratories, Inc.). Enrichment and depletion of DNA were calculated using the Δ*C*_*t*_ of the whole cell compared to the mitochondrial isolate.

## ***CellTiter-Glo***^®^

Cell viability and ATP concentration were assessed using the Promega CellTiter-Glo^®^ luminescent cell viability assay (Promega, #G7572) according to the manufacturer’s instructions. Briefly, samples and an ATP standard curve (0–10 μM) were added to a 96-well plate and combined with the CellTiter-Glo^®^ substrate. Plates were incubated for 10 min, followed by a luminescence reading using a Synergy H1 microplate reader (BioTek Instruments, Inc., Winooski, Vermont, USA). The sample’s relative luminescent unit was compared to the ATP standard curve to quantify total ATP within each sample, which is proportionate to cell viability.

ATP concentration was used to assess functionality of isolated mitochondria. Mitochondria were isolated from 2 × 10^7^ HEK293 cells and resuspended in 1000 μl of respiration buffer. Mitochondria were plated at 50 μl/well of live or liquid nitrogen inactivated (LN2) and incubated with the CellTiter-Glo substrate to evaluate mitochondrial ATP concentration. Furthermore, mitochondrial ATP concentration was measured at 0, 120, 180, and 240 min post-isolation.

### Biolog mitochondrial function assay

Biolog’s tetrazolium redox dye was used to measure the active oxidative phosphorylation capacity of the isolated mitochondria with and without malate treatment. Mitochondria were isolated from 2 × 10^7^ HEK293 cells and plated at 30 μl/well according to the manufacturer’s specifications. Mitochondria were treated with 4 mM malate to activate complex I of the electron transport chain. Mitochondria were incubated with the redox dye, which acts as a terminal electron acceptor and turns purple upon reduction at the distal end of the electron transport chain. Absorbance was read every 5 min for 3 h total using a Synergy H1 microplate reader (BioTek Instruments, Inc., Winooski, Vermont, USA) at 570 nm and 750 nm.

### Flow cytometry for mitochondria quantification and quality control

Flow cytometry was performed on the mitochondrial isolates using a Beckman Coulter CytoFLEX (blue (488 nm) and red laser (638 nm) configuration with volumetric sensor, Brea, California, USA; used for HEK293, MDA-MB-231, and MCF7 cell lines) and Cytek Aurora (violet (405 nm), blue (488 nm), and red laser (640 nm) configuration with volumetric sensor; Fremont, California, USA; used for iPSCs and CO) to evaluate mitochondrial viability by co-staining with MitoTracker™ Red CMXRos (MTviable; Invitrogen, #M7512) and Green FM (MTall.Green; Invitrogen, #M7514). Compensation matrices or spectral unmixing were performed on the day of each experiment. The combination of these dyes allows us to distinguish viable and non-viable mitochondria, since MTviable is mitochondrial membrane potential (MMP) dependent while MTall.Green is MMP independent [33]. Briefly, isolated mitochondria were stained with 230 nM MTviable and/or MTall.Green for 5 min at 37 °C and washed 3 times with PBS. Samples were kept in the dark and on ice until flow cytometry was performed. Using the cytoFLEX flow cytometer, mitochondrial events were identified by the fluorescent intensity of MTall.Green, since it is not dependent on MMP and will not fluoresce in an aqueous solution. MTall.Green positive events were then gated based on their forward vs side scatter (FSC-A, SSC-A). This gating was then applied to all experiments. Events within this population were plotted as a histogram by fluorescent intensity of MTviable. Using the Cytek Aurora flow cytometer, events were first gated by FSC-A vs SSC-A, and then plotted with MTviable on the y-axis and MTall.Green on the *x*-axis. The concentration of co-stained events was then calculated using the instrument’s volumetric sensor and acquisition software. When mitochondria were isolated from HEK293-mitoDsRed2 cells, MitoTracker™ DeepRed (MTall.DeepRed; Invitrogen, #M22426) was used due to the overlap of the fluorescent emission of MTviable and mitoDsRed2. The flow cytometry results were analyzed using FlowJo™ v10.8 Software (BD Life Sciences) [48].

### Western blot

Mitochondrial enrichment and purity were evaluated through western blotting for thymidine kinase (TK1) (1:500, Invitrogen, #MA5-32,513) and HEK293 marker, E1A (1:1000, Sigma-Aldrich, #05-599) [34]. Samples were prepared in Laemmli buffer and loaded at 35 μg/lane onto a 10% acrylamide gel (#4561034, Bio-Rad Laboratories Ltd., Mississauga, ON, Canada) and then transferred to a polyvinylidene difluoride (PVDF) membrane. The membrane was then blocked with 5% skim milk in TBS-T for 30 min at room temperature, then incubated with the primary antibody overnight at 4 °C. Following the primary antibody, membranes were incubated for 1 h at room temperature with an HRP-conjugated secondary antibody (1:2000; Anti-rabbit IgG, Cell Signaling Technologies, #7074; Anti-mouse IgG, Cell Signaling Technologies, #7076). Membranes were then visualized with ECL (GE Healthcare, RPN2106) and Versa Doc (Bio-Rad Laboratories Ltd., Mississauga, ON, Canada). GAPDH was used as a loading control (1:10,000, Invitrogen, #39-8600). Following visualization, membranes were either dried for storage or stripped using a guanidine stripping buffer.

### Statistical analysis

Results for nDNA, mtDNA, and TEM quantification were analyzed using GraphPad Prism version 9.1.0 for Windows (GraphPad Software, San Diego, California USA, www.graphpad.com). Shapiro–Wilk and Brown–Forsythe tests were used to assess normality and homogeneity of variance, respectively. One-way ANOVA, Welch’s ANOVA, and two-tailed t-test were used to analyze the results where appropriate. Tukey’s and Dunnett T3 multiple comparisons tests were performed with one-way and Welch’s ANOVA, respectively.

## Results

### Mitochondrial transplant—cancer cell lines

**Fig. 1:**
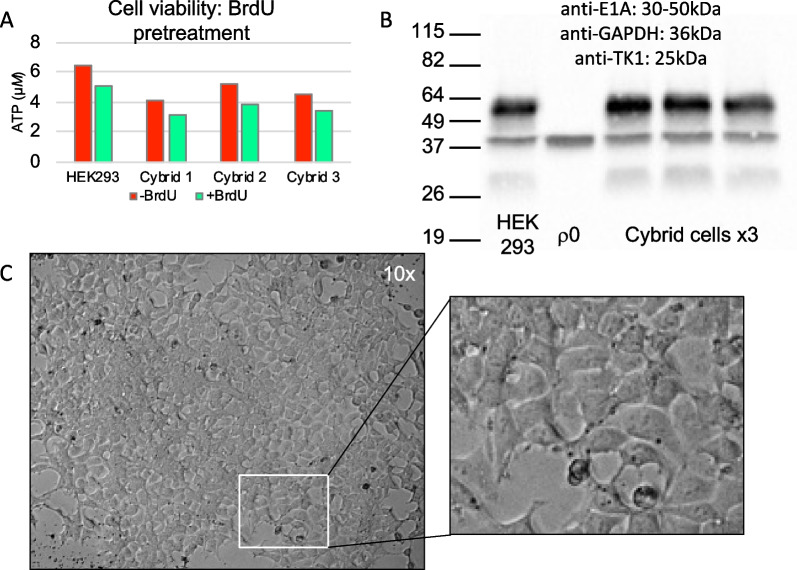
Confirmation of whole cell contamination in mitochondrial isolate. **A** Assessment of ATP via CellTiter-Glo with 48-h pretreatment of BrdU. **B** Western blot of E1A and thymidine kinase in HEK293, ρ0, and post-mitochondrial transplant cells. Uncropped western blots can be found in Additional file 1: Fig. S1. **C** 8 days after plating mitochondria isolate with no host cells

We tested the mitochondrial transplant using HEK293 cells as a mitochondrial donor and osteosarcoma 143B (TK-) ρ0 cells as a host using the mitochondrial isolation method from Preble et al*.* [19]. Following expansion of the mitochondrial transplant cells, we then treated the cells with bromodeoxyuridine (BrdU) to confirm the cell identity as a true cybrid cell line. However, upon treating the cybrid cells with BrdU, we found that the treatment reduced cellular viability indicating a lack of BrdU resistance (Fig. 1a). We then performed a western blot to confirm the absence of TK1 and the HEK293 marker, E1A [34]. The presence of both TK1 and E1A in the western blots confirmed that the resulting cells were not cybrids and were HEK293 cells (Fig. 1b; uncropped western blots can be found in Additional file 1: Fig. S1). Experiments depicted in Fig. 1a and b were replicated in a separate independent experiment (Additional file 1: Fig. S2). We further confirmed the results by plating isolated mitochondria from HEK293 cells without any host cells. After 8 days, we imaged the samples and found cells were beginning to repopulate the well (Fig. 1d), confirming the presence of whole cell contamination in our mitochondrial isolate.

### Optimizing mitochondrial isolation—cancer cell lines

**Fig. 2:**
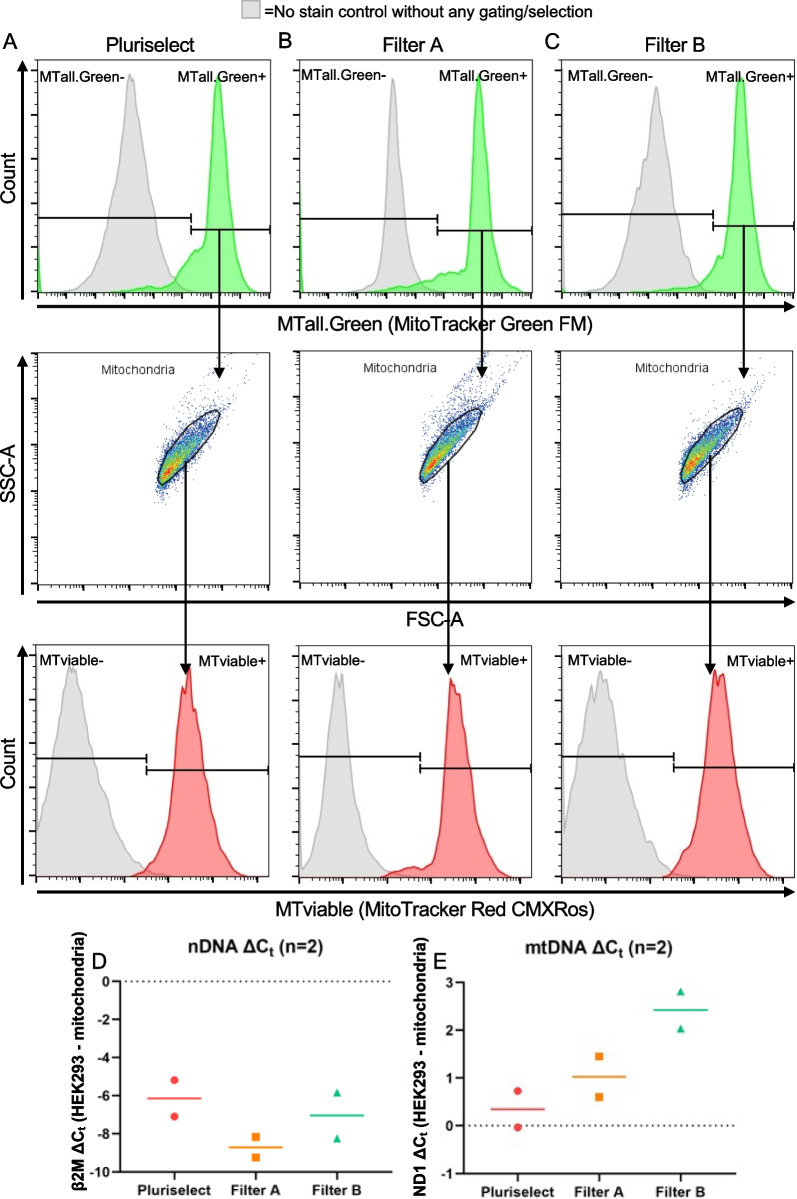
Results of mitochondrial isolation in HEK293 cells comparing 5-μm filter types. **A**–**C** Flow cytometry results of isolated mitochondria from pluriselect, filter A, and filter B. Events were first gated by fluorescent intensity of MTall.Green-A, followed by FSC-A vs SSC-A. Lastly, mitochondrial viability was confirmed by MTviable staining. Experiments were performed on Beckman Coulter CytoFLEX. **D** and **E** Δ*C*_*t*_ of HEK293 whole cells and mitochondrial isolates for nDNA depletion and mtDNA enrichment, respectively

We tested the mitochondrial isolation method with new 5-μm filters to mitigate the whole cell contamination. Figure 2a–c shows the flow cytometry results of the mitochondrial isolates from HEK293 cells. We first confirmed that events were mitochondria indicated by the positive MTall.Green staining, which stains all mitochondria and is not fluorescent in an aqueous solution or dependent on MMP. Furthermore, mitochondria were viable, as indicated by the positive stain of MTviable, which stains mitochondria and is dependent on MMP. Additionally, qPCR data from all show an enrichment of mtDNA and depletion of nDNA compared to HEK293 cells (Fig. 2d, e). Interestingly, filter A had the highest nDNA depletion compared to filter B and pluriselect filters, whereas filter B maintained the highest mtDNA. These experiments were also repeated in the MDA-MB-231 and MCF7 cancer cell lines with similar results (Additional file 1: Table S1). One-way ANOVA and t-test were used to analyze changes in nDNA and mtDNA but found no significance, which could be related to the small sample size and therefore should be taken with caution.

**Fig. 3:**
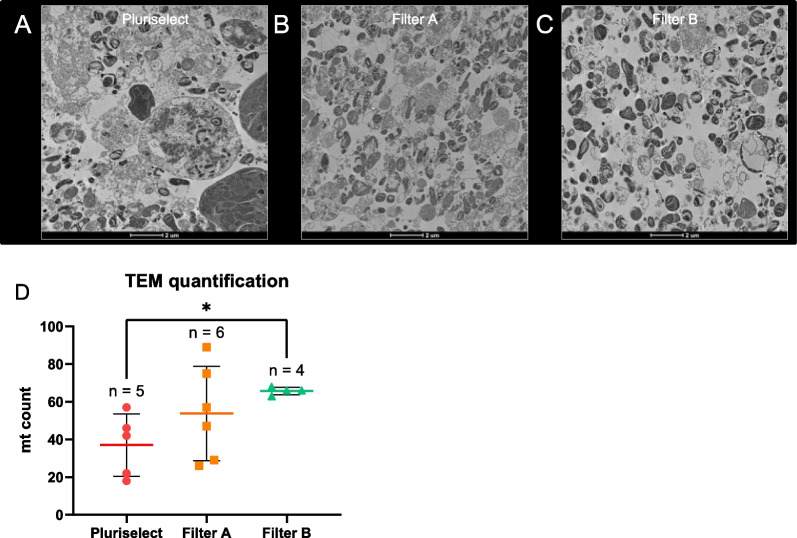
TEM images of isolated mitochondria from HEK293 cells using each brand of filter. **A**–**C** Mitochondria isolated with 5-μm pluriselect filter, filter A, and filter B, respectively. (4300×). Scale bar, 2 μm. **D** Semi-automated quantification of mitochondria in TEM images. n = number of images used for quantification. *: *p* < 0.05, Welch’s ANOVA with Dunnett T3 multiple comparisons

Once we confirmed isolated mitochondria from each brand of filter maintained their mitochondrial membrane potential, we isolated mitochondria from HEK293 cells for TEM (Fig. 3a–c). As expected from our earlier experiments (Fig. 1d), we can see whole cell contamination in images of mitochondria isolated using the 5-μm pluriselect filters. Mitochondrial preparations from filter A and filter B both have no whole cell contamination. Furthermore, we found that isolated mitochondria using filter B had significantly increased mitochondrial count compared to pluriselect filters (Fig. 3d; Welch’s ANOVA: *p* < 0.05), which is consistent with the mtDNA enrichment shown in Fig. 2e. Mitochondria isolated with filter B appear to have less cellular debris and mitochondrial clumping compared to mitochondria from filter A. Therefore, we selected filter B for further testing due to its higher quality and yield of mitochondria.

**Fig. 4:**
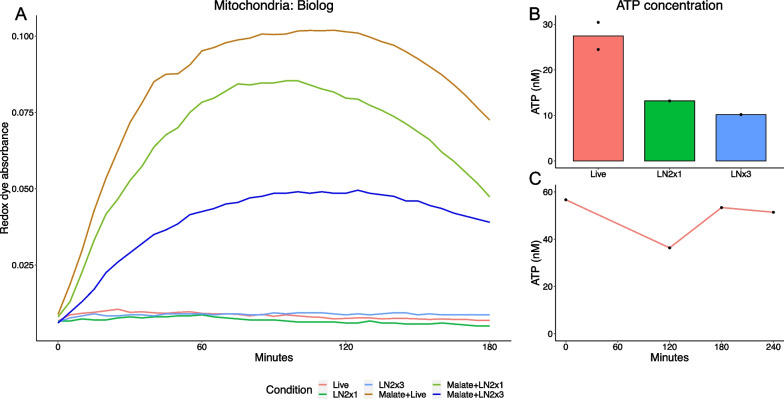
Confirmation of functional oxidative phosphorylation in mitochondria isolated from HEK293 cells. **A** Rate of reduction of biolog’s tetrazolium based in isolated mitochondria. Mitochondria were measured at baseline (live) and malate activated, as well as LN2 inactivated with and without malate treatment. **B** ATP concentration of live and LN2 inactivated mitochondria. **C** Time course of ATP concentration at 0, 120, 180, and 240 min in live mitochondria. LN2×1: one freeze–thaw cycle; LN2×3: three freeze–thaw cycles

Biolog’s tetrazolium redox dye, which is reduced at the distal end of the electron transport chain, was used to confirm the functionality of the isolated mitochondria. This assay is designed and validated for use in both whole cells and isolated mitochondria to assess mitochondrial metabolism of various substrates. Mitochondria treated with malate have a drastically higher rate of dye reduction compared to untreated mitochondria, indicating increased rate of electron flow through the electron transport chain (Fig. 4a). Furthermore, mitochondria lose functionality with repeated LN2 freeze–thaw cycles. Mitochondrial electron flow peaks at 60min and is stable until 150 min, followed by a slight decrease. This is consistent with findings from Doulamis and McCully [29] that demonstrated mitochondrial oxygen consumption was steady until 120 min. We further confirmed functionality by measuring ATP in the live mitochondria compared to the LN2 inactivated mitochondria. Total mitochondrial ATP was reduced by ~ 60% after freeze–thaw cycles in LN2 (Fig. 4b). Additionally, we assessed mitochondrial ATP at 0, 120, 180, and 240 min and found ATP concentration was steady throughout the time points, with a slight decrease at 120 min (Fig. 4c). Taken together, these data confirm that the isolated mitochondria are functional.

### Validating mitochondrial isolation—induced pluripotent stem cells (iPSCs)

**Fig. 5:**
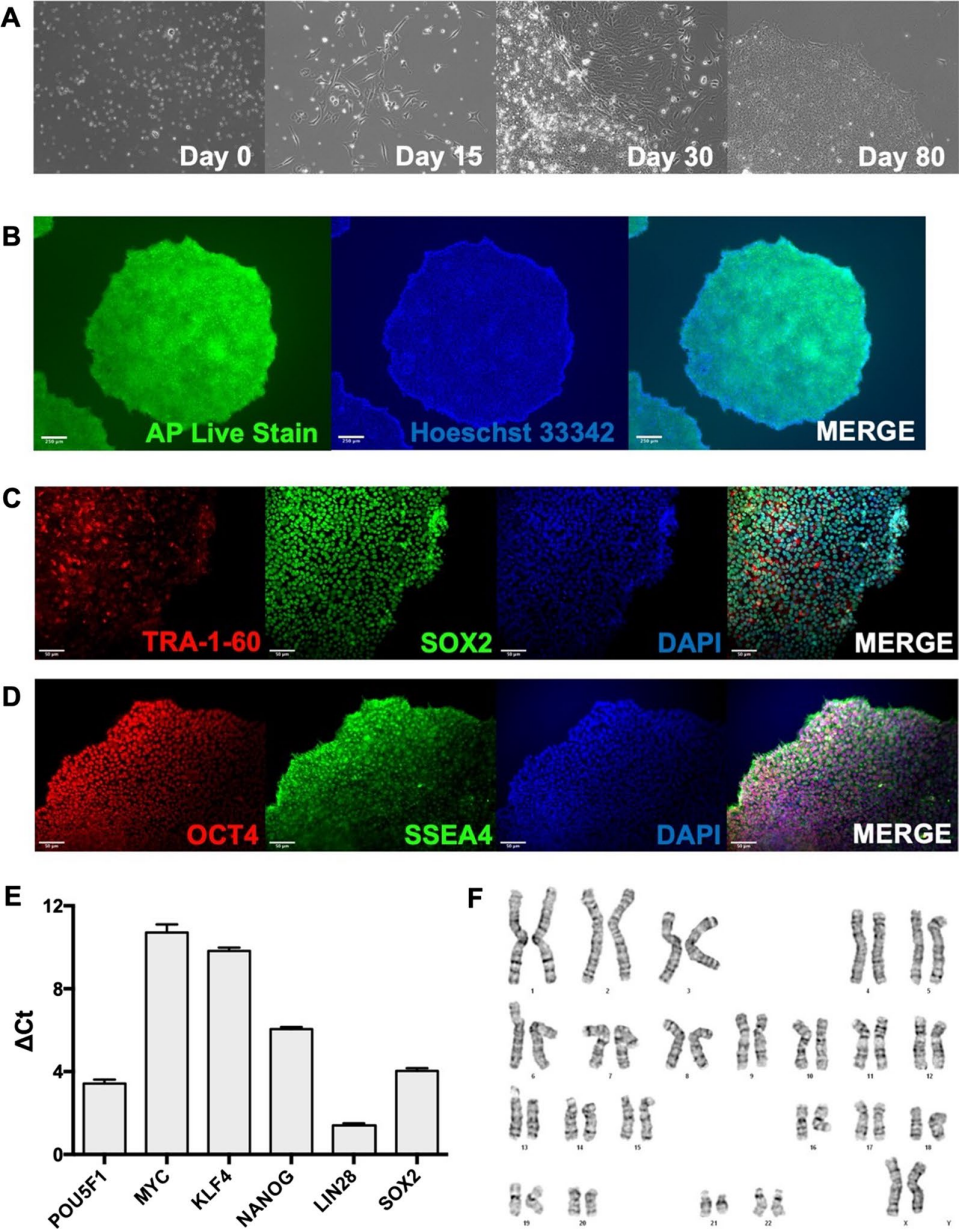
Full characterization of iPSC derived from commercially purchased human PBMCs. **A** Light microscopy with phase contrast images using of PBMC upon recovery (Day 0); fibroblast-like morphological transformation at Day 15 post-transfection; iPSC colony formation surrounded by differentiated cells at Day 30; iPSC colony with minimal differentiated cells at Passage 5 (Day 80). Images acquired using 10X objective lens. **B** Alkaline phosphatase (AP) Live cell staining of iPSC colonies with Hoechst 33,342 nucleus staining. Images acquired using 4X objective lens. Scale bar, 250 μm. **C** Immunocytochemistry of TRA-1-60 and SOX2 with DAPI nucleus staining of iPSC colonies. Scale bar, 50 μm. **D** Immunocytochemistry of OCT4 and SSEA4 with DAPI nucleus staining of iPSC colonies. Scale bar, 50 μm. **E** Gene expression of pluripotency markers (POU5F1, MYC, KLF4, NANOG, LIN28 and SOX2) expressed in average ΔC_*t*_. ΔC_*t*_ calculated by determining the difference between target gene and housekeeping gene (average C_*t*_ of GAPDH and ACTB). Error bars represent standard deviation. **F** Karyotype image representing normal karyotype of 46, XX. All acquired images represent entire field of view with no cropping done to the image. No digital zoom applied to any of the images

Characterization of iPSC derived from ATCC-PBMCs iPSC by karyotype analysis and pluripotency marker expression appear normal (Fig. 5). G-band analysis shows normal 46 female chromosomes indicating that no chromosomal alteration occurred during the iPSC generation. Staining of alkaline phosphatase and immunofluorescence of pluripotency markers were positive. Furthermore, mRNA expression of pluripotency markers was upregulated confirming the generated iPSCs were pluripotent.

**Fig. 6:**
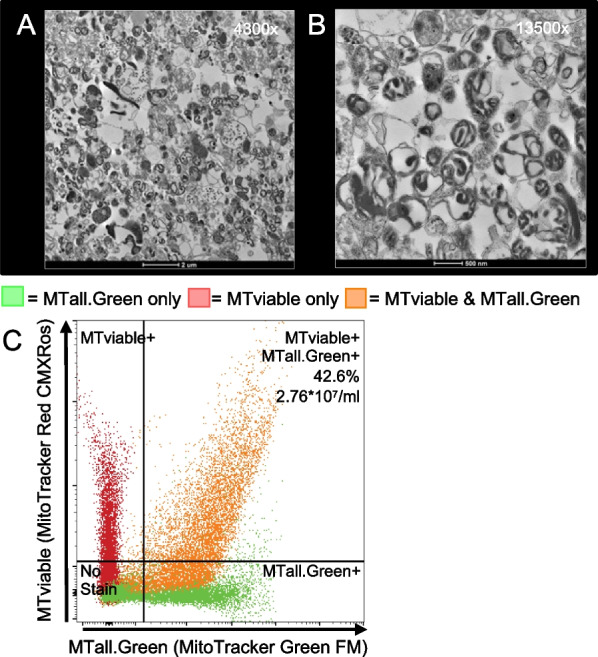
TEM images of mitochondria isolated from iPSCs using filter B. **A** and **B** TEM images at 4300× and 13,500×, respectively. **C** Flow cytometry of isolated mitochondria. Green and red represent single stained controls. Orange represents co-stained sample

After removing the whole cell contamination and optimizing the mitochondrial isolation in cancer cell lines, we validated the protocol in ATCC-derived iPSCs through TEM and flow cytometry (Fig. 6). Mitochondrial morphology from these samples is indicative of an immature mitochondrion compared to the TEM images of the mitochondria from cancer cell lines (Fig. 3a–c). However, this morphology is consistent with previous findings from iPSCs [21]. The purity of the isolated mitochondria is reduced compared to the cancer cell lines. Furthermore, the flow cytometry results (Fig. 6c) are consistent with the TEM images, with 28.5% of events not staining MTall.Green or MTviable (i.e., debris) and an additional 28.2% staining only with MTall.Green (i.e., non-viable mitochondria; Fig. 6c). From 4 × 10^7^ iPSCs, we isolated 4.14 × 10^7^ viable mitochondria (2.76 × 10^7^/ml × 1.5 ml total volume of mitochondria; Fig. 6c). Lastly, the qPCR shows the mitochondria isolated from iPSCs with filter B have a slight enrichment in mtDNA (Δ*C*_*t*_ = 0.19) and a depletion of nDNA (Δ*C*_*t*_ = − 5.53).

### Proof-of-principle experiment: mitochondrial transplant in cerebral organoids (CO)

**Fig. 7:**
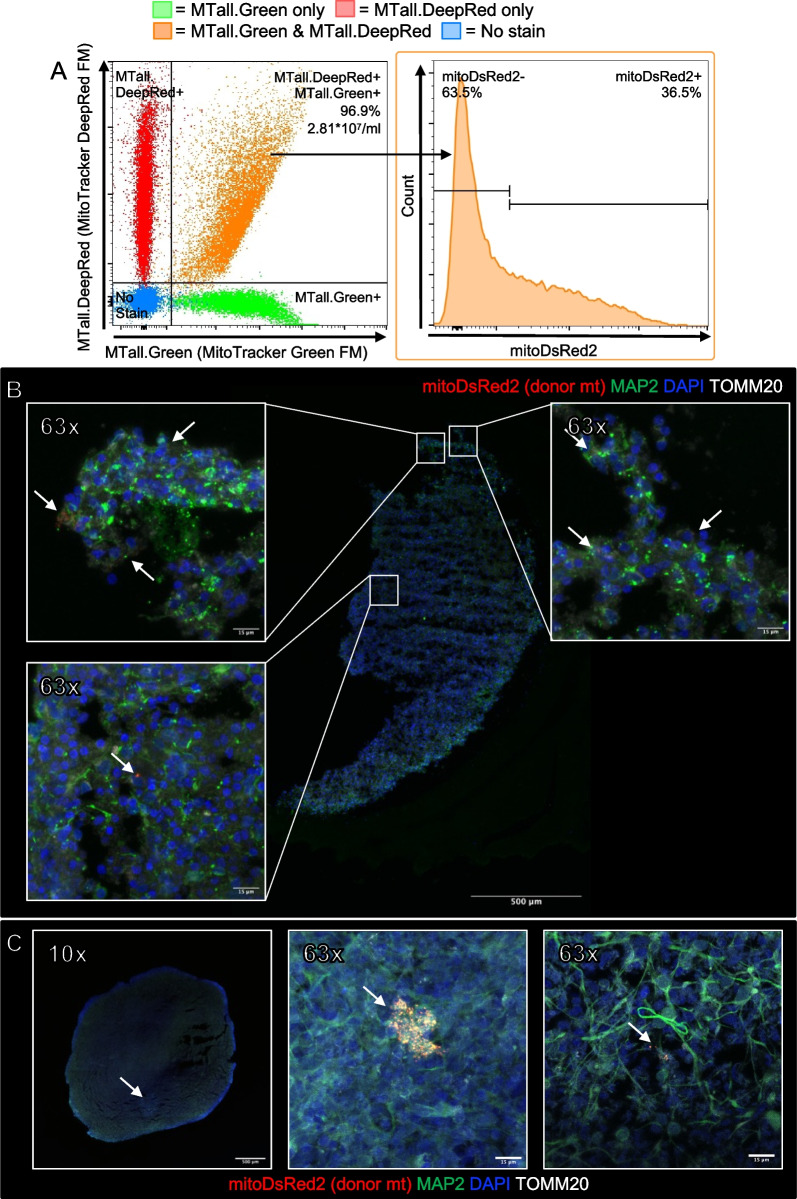
Proof-of-principle mitochondrial transplant in H9 embryonic CO. **A** Flow cytometry of the mitochondrial isolate to determine final mitochondrial concentration. Percentage of DsRed2-tagged mitochondria that co-stain with MTall.Green and MTall.DeepRed. **B** 3.74 × 10^6^ (133ul) isolated viable mitochondria were transplanted into 4-month-old H9 embryonic CO. Arrows indicate DsRed2-tagged donor mitochondria. 10× tile scan of whole organoid slice. Scale bar, 500 μm. Inset panels are DsRed2-tagged donor mitochondria engulfed in CO cells. Scale bar, 15 μm. Arrows indicate DsRed2-tagged donor mitochondria. **C** Replicate of mitochondrial transplant in H9 embryonic CO with DsRed2-tagged mitochondria. Arrows indicate DsRed2-tagged donor mitochondria. 10× scale bar, 500 μm; 63× scale bar, 15 μm

We performed a proof-of-principle mitochondrial transplant in a 4-month-old female H9 embryonic CO (full characterization in Duong et al. [21] and Sivitilli et al. [22]). DsRed2-tagged mitochondria were isolated using filter B from HEK293-mitoDsRed2 cells and transplanted by volume (133 μl of mitochondria) through co-incubation with 400-μm CO slices for 2 h at 37 °C. We had a final mitochondrial concentration of 2.81 × 10^7^ mitochondria/ml, of which 36.6% were tagged with mitoDsRed2 (Fig. 7a, b). Therefore, a total of 3.74 × 10^6^ mitochondria were transplanted into the CO. CO were fixed at 24 h post-transplant for immunofluorescence and labeled with antiTOMM20, a universal mitochondrial marker, and antiMAP2, a neuronal cell marker. In Fig. 7c, we see co-localization of the DsRed2-tagged donor mitochondria with MAP2 and TOMM20 indicating successful uptake and penetrance into an inner slice of the CO. This experiment was replicated with a 200-μm sliced 4.5-month-old female H9 embryonic CO that was transplanted with 200ul of isolated mitochondria. We replicated the immunofluorescence and confirmed the co-localization of the DsRed2-tagged donor mitochondria with MAP2 and TOMM20 (Fig. 7d).

## Discussion

The development of autologous mitochondrial transplants might offer a novel therapeutical approach for chronic and rare diseases associate with mitochondrial dysfunction. Nonetheless, prior to evaluating its therapeutic efficacy, we must be able to rapidly and effectively isolate mitochondria and perform the transplant in vitro. In this study, we adapted the mitochondrial isolation protocol from Preble et al. [19], which was previously used in pediatric patients with congenital heart disease [16], for use in a cellular model. The differential filtration method created by Preble et al*.* [19] is advantageous to the more common differential-centrifugation as it removes the need for multiple centrifugation steps reducing the mitochondrial isolation time. This is important because isolated mitochondria are only viable for a short period, therefore, a quicker isolation allows a longer working time with the isolated mitochondria [19, 29]. While testing the protocol, we found that the 5-μm pluriselect filters from the original protocol led to whole cells contaminating the final mitochondrial isolate. We began testing the mitochondrial isolation method with 5-μm filter A and filter B to remove the whole cell contamination. We found that the 5-μm filter B provided the highest quality and purity of live isolated mitochondria, and therefore, was selected for further testing. Notably, we use a flow cytometry method to count live mitochondria instead of using a Coulter counter enabling precision for experiments. We then performed a proof-of-principle mitochondrial transplant in H9 embryonic COs using the filter B to confirm the mitochondrial transplant and found that mitochondria successfully penetrate the COs cells. With the method proposed here, we are now capable of efficiently isolating live and pure mitochondrial from cellular models (such as iPSC or COs) opening venue to investigate possibilities of autologous mitochondrial transplant using cellular sources and not only tissue.

There are several ways to confirm cybrid cell identity following generation, from simple methods, such as selective agents, to nDNA and mtDNA sequencing [35]. In our testing, we selected against unfused osteosarcoma 143B(TK-) ρ0 host cells by removing the uridine supplementation required for their growth. Supplementing the ρ0 cells with uridine allows pyrimidines to be synthesized through the salvage pathway, rather than the oxidative phosphorylation-dependent de novo pathway [36]. By removing uridine supplementation, unfused ρ0 cells are unable to survive as cells cannot synthesize pyrimidines due to the lack of oxidative phosphorylation [36]. Furthermore, we selected against whole HEK293 cells using the thymidine analogue, BrdU, which is phosphorylated by thymidine kinase and integrated into replicating nDNA leading to cell death [13, 37]. Osteosarcoma 143B(TK-) ρ0 cells are resistant to BrdU treatment due to the absence of thymidine kinase [13]. Cybrid cells are capable of surviving both these selective agents as they are capable of oxidative phosphorylation to support pyrimidine synthesis from the donor mitochondria, while maintaining the BrdU resistance from the osteosarcoma 143B(TK-) nuclear background.

During our testing of the mitochondrial transplant using donor mitochondria from HEK293 cells and osteosarcoma 143B(TK-) ρ0 host cells, it initially appeared the transplant was successful. However, after treating the resulting cells with BrdU, the cells lacked the expected resistance of the osteosarcoma 143B(TK-) ρ0 cells, which was supported by western blot showing TK1 protein expression in the resulting cells. The protein expression of TK1 indicates the nuclear origin of the cells did not originate from the osteosarcoma 143B(TK-) ρ0 cells as expected. We used the HEK293 marker, E1A, to confirm the nuclear origin of the cells is from the donor HEK293 cells. E1A is encoded by an adenovirus gene fragment inserted into chromosome 19 from the adenovirus used to immortalize the HEK293 cell line [38]. Thus, the E1A protein can be used as a marker for HEK293 cells allowing us to confirm the identity of the resulting cells as HEK293. This set of experiments were replicated on two sets of independent mitochondrial transplant cells with similar results (Additional file 1: Fig. S1). By combining the use of cell specific selective agents and protein expression unique to each parent cell line, we were able to confirm the presence of whole cell contamination in our mitochondrial isolate.

Whole cell contaminants in the mitochondrial isolate can lead to significant adverse reactions and long-term effects in patients if the contaminating cells continue proliferating [39]. To remove this unwanted potential side effects, we began optimizing the mitochondrial isolation protocol to remove the whole cell contamination to proceed with developing an effective mitochondrial transplant. Cancer lines were used to optimize the mitochondrial isolation protocol due to their ease of growth and expansion. We focused on the 5-μm filter as it is the last filtration step and to avoid additional centrifugation steps to maintain the speed of the filtration-based method. It was important to repeat quality controls with the new filters to confirm that isolated mitochondria were viable and pure. We stained isolated mitochondria from each 5-μm filter with MTviable and MTall.Green to confirm viability. This allowed us to quickly identify all mitochondrial events with MTall.Green and further classify these events based on viability from MTviable in each 5-μm filter type. It was essential to confirm that mitochondria isolated from each 5-μm filter were viable and respiration competent, as McCully et al. [40] found that only the transplant of viable mitochondria were able to produce a therapeutic effect.

It is necessary to transplant live and pure mitochondrial isolates to avoid immune reaction and adverse events. Mitochondrial stress and the release of mtDNA, which is recognized as a damage-associated molecular pattern (DAMP), can trigger the activation of the NLRP3-inflammasome leading to an immune reaction [41]. We used qPCR to compare mtDNA and nDNA enrichment/depletion between all samples normalized to DNA extracted from whole cells. This allowed us to individually evaluate the presence of nDNA contaminants, which can trigger an immune reaction [42], and mtDNA enrichment between all samples rather than combining to estimate the relative mtDNA copy number. Although filter B had lower nDNA depletion compared to the filter A, filter B had higher mtDNA enrichment, which is consistent with the TEM quantification. Furthermore, TEM was used to determine other cellular organelle contamination, most commonly endoplasmic reticulum, lysosome, and peroxisome [18]. Mitochondria isolated with filter B were higher quality compared to the filter A with less mitochondrial clustering and cellular debris. Taken together, both the filter A and filter B can be used to isolate viable mitochondria without whole cell contamination. We opt to proceed forward with filter B due the higher quality and enrichment of mitochondria.

After optimizing the mitochondrial isolation in cancer cell lines, we validated the method in human PBMC-derived iPSCs. The use of flow cytometry allowed us to accurately quantify viable, live, mitochondria by counting only co-stained mitochondrial events rather than using a particle counter, which recognizes based on size characteristics [42]. This counting method provides a unique advantage by allowing us to be more consistent across mitochondrial isolations and transplants by accounting for variation in mitochondrial viability and quality. Using this method, we identified that 42.6% of mitochondrial events were viable mitochondria with the remainder being non-viable mitochondria and debris. This was consistent with the TEM images of the isolated mitochondria from iPSCs, which showed reduced purity compared to HEK293 mitochondrial images. This could be related to several factors including initial cell lysis and final mitochondrial pelleting speed, as well as other cell-type specific effects. Specifically, mitochondrial morphology and dynamics are altered in iPSCs to promote glycolysis over OXPHOS compared to HEK293 and other differentiated cell lines [21, 43]. iPSC mitochondria are immature and globular compared to mature mitochondria, which form elongated networks to support oxidative phosphorylation [21, 43]. This morphology is consistent with what we see in our TEM images of isolated mitochondria from iPSC. Taken together these changes in mitochondria morphology and dynamics may influence the mitochondrial yield and potentially final pelleting speed. Additionally, immature mitochondria are much more perinuclear, whereas mature mitochondria in differentiated cells are dispersed in the cytoplasm, which also may affect the purity and quality of mitochondria. Therefore, it may be necessary to explore the technique for mitochondrial transplant using differentiated cell lines that have mature mitochondria.

The proof-of-principle mitochondrial transplant of 3.74 × 10^6^ mitochondria in H9 embryonic CO indicates that the new mitochondrial isolation protocol does not affect uptake of mitochondria. We saw penetration of donor mitochondria into an internal slice of the CO. In this experiment, due to the overlap of the fluorescent emission of mitoDsRed2 and MTviable, it was not possible to the obtain the concentration of viable mitochondria. However, by co-staining with MTall.DeepRed and MTall.Green we could obtain an accurate mitochondrial concentration with these independent mitochondrial stains. This experiment has been replicated on 2 independent mitochondrial transplants. Previous work by Masuzawa et al. [27] found that mitochondrial transplant in cardiomyocytes significantly increased oxygen consumption rate and improved long-term recovery in rabbits at 4 weeks. Furthermore, cybrid generation with ρ0 cells, which lack all oxidative phosphorylation capacity, has been extensively shown to incorporate donor mitochondria and restore oxidative phosphorylation [36, 44–47]. However, the clinical use of mitochondrial transplantation focuses on functional improvement of tissue endpoints. Therefore, future experiments will not use DsRed2-tagged mitochondria allowing us to quantify the concentration of viable donor mitochondria with MTviable and MTall.Green co-staining to characterize long-term integration and functional effects of mitochondrial transplantation in CO.

## Conclusions

Taken together, we adapted and optimized the differential filtration-based mitochondrial isolation protocol developed by Preble et al. [19] for use in cells to obtain a high-quality and pure mitochondrial isolate. We identified the presence of whole cell contaminants when mitochondria were isolated from HEK293 cells using the 5-μm pluriselect filters, which was confirmed by western blot and TEM. We identified the optimal filter that yielded the highest quality and quantity of isolated mitochondria by evaluating the mitochondrial isolation with new 5-μm filters in multiple cell lines. We further validated the use of the adapted mitochondrial isolation protocol in iPSC and performed a proof-of-principle mitochondrial transplant in H9 embryonic CO with DsRed2-tagged mitochondria. Future experiments are required to evaluate the long-term integration of donor mitochondria and the effects on mitochondrial function. Furthermore, DAMP recognition and inflammatory response should be reduced with the new 5-μm filters due to improved mitochondrial purity. However, further experiments are required to assess the safety profile of mitochondrial transplants with the new 5-μm filters.

### Supplementary Information

**Additional file 1**. **Fig S1:** Uncropped western blots from Fig. 1B of thymidine kinase and E1A in HEK293, r0, and post-mitochondrial transplant cells. **Fig S2:** Biological replicate of mitochondrial transplant of mitochondria isolated from HEK293 cell and transplanted to rho-0 (r0) cells. **A**) Assessment of ATP via cell titer glo with 48hr pretreatment of BrdU. **B & C**) Western blot of thymidine kinase and E1A in HEK293, r0, and post-mitochondrial transplant cells. Samples were prepared in laemmli buffer and loaded at 50μg/lane onto a 10% acrylamide gel and blotted for thymidine kinase (TK1; 1:1000) and HEK293 marker, E1A (1:1000). GAPDH (1:10000) was used as a loading control. **Table S1**: ΔCt of HEK293 whole cells and mitochondrial isolates for nDNA depletion and mtDNA enrichment, respectively.

## Data Availability

The datasets used and/or analyzed during the current study are available from the corresponding author on reasonable request.

## Abbreviations

iPSC: Induced pluripotent stem cells
ATP: Adenosine triphosphate
OXPHOS: Oxidative phosphorylation
mtDNA: Mitochondrial DNA
nDNA: Nuclear DNA
LHON: Leber’s Hereditary Optic Neuropathy
MERRF: Myoclonic Epilepsy with Ragged Red Fibers
MELAS: Mitochondrial myopathy, encephalopathy, lactic acidosis, and stroke-like episodes
CO: Cerebral organoid
mitoDsRed2: DsRed2-Mito-7
PBMC: Peripheral blood mononuclear cells
AP: Alkaline phosphatase
MTviable: MitoTracker™ Red CMXRos
MTall.Green: MitoTracker™ Green FM
MTall.DeepRed: MitoTracker™ Deep Red
TEM: Transmission electron microscopy
TK1: Thymidine kinase
BrdU: Bromodeoxyuridine
DAMP: Damage-associated molecular pattern
