# Supplementary material for: Optimization of differential filtration-based mitochondrial isolation for mitochondrial transplant to cerebral organoids
Source: Stem Cell Res Ther. 2023 Aug 15;14:202. doi: 10.1186/s13287-023-03436-y (PMC10426050; doi:10.1186/s13287-023-03436-y)
Supplement: Supplementary file 1 — Additional file 1. Fig S1: Uncropped western blots from Fig. 1B of thymidine kinase and E1A in HEK293, r0, and post-mitochondrial transplant cells. Fig S2: Biological replicate of mitochondrial transplant of mitochondria isolated from HEK293 cell and transplanted to rho-0 (r0) cells. A) Assessment of ATP via cell titer glo with 48hr pretreatment of BrdU. B & C) Western blot of thymidine kinase and E1A in HEK293, r0, and post-mitochondrial transplant cells. Samples were prepared in laemmli buffer and loaded at 50μg/lane onto a 10% acrylamide gel and blotted for thymidine kinase (TK1; 1:1000) and HEK293 marker, E1A (1:1000). GAPDH (1:10000) was used as a loading control. Table S1: ΔCt of HEK293 whole cells and mitochondrial isolates for nDNA depletion and mtDNA enrichment, respectively. [file 13287_2023_3436_MOESM1_ESM.pdf]

Supplementary information

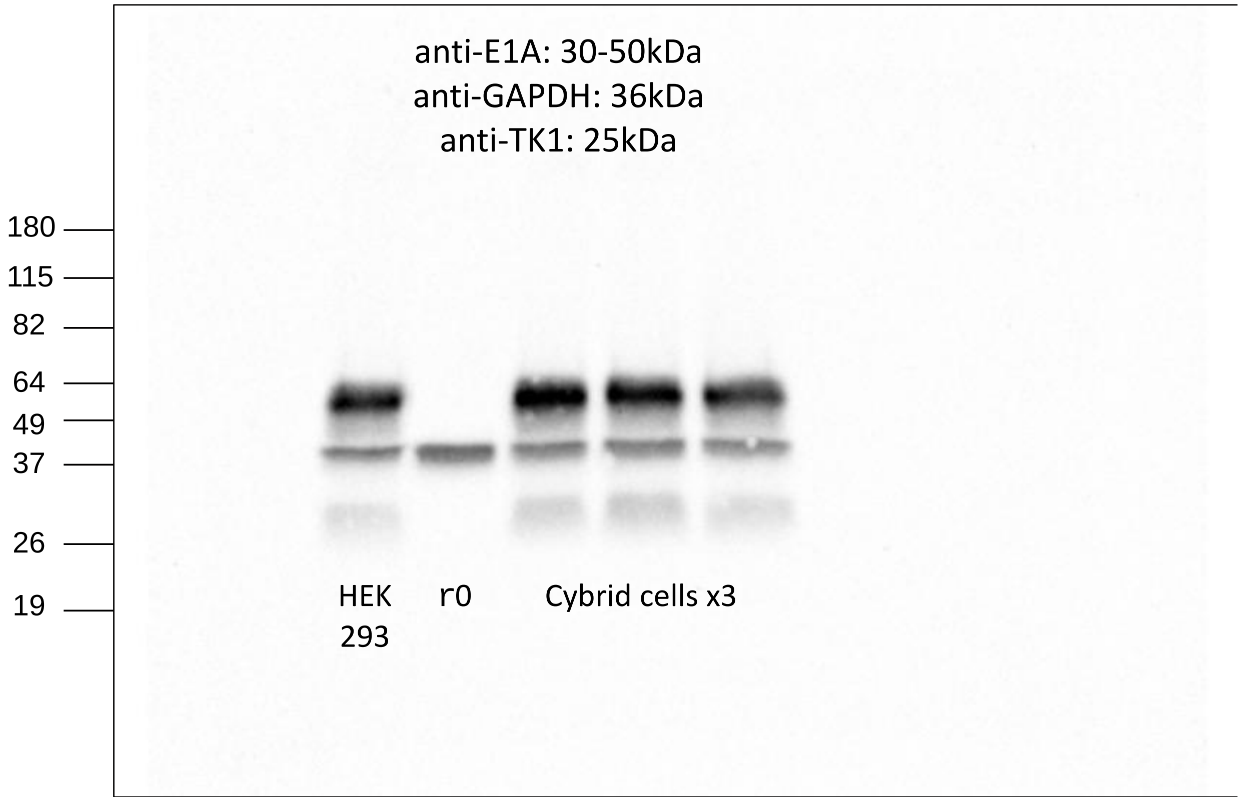

Supplementary Fig 1. Uncropped western blots from Fig. 1B of thymidine kinase and E1A in HEK293, ρ0, and post-mitochondrial transplant cells.

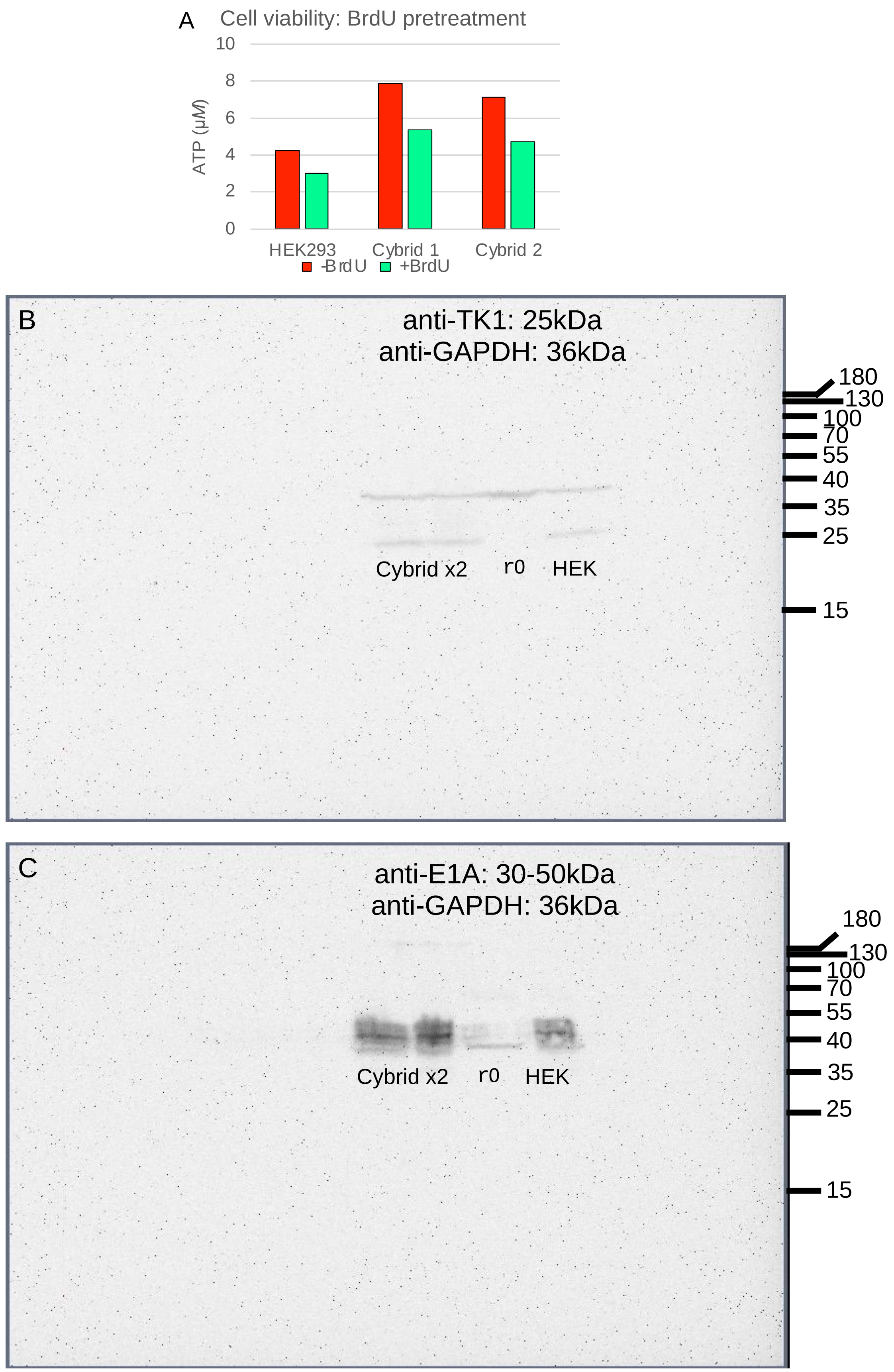
A
Cell viability: BrdU pretreatment
10
8
6
4
2
0
ATP (µM)
HEK293
Cybrid 1
Cybrid 2
-BrdU
+BrdU
B
anti-TK1: 25kDa
anti-GAPDH: 36kDa
Cybrid x2
r0
HEK
180
130
100
70
55
40
35
25
15
C
anti-E1A: 30-50kDa
anti-GAPDH: 36kDa
Cybrid x2
r0
HEK
180
130
100
70
55
40
35
25
15

Supplementary Fig 2. Biological replicate of mitochondrial transplant of mitochondria isolated from HEK293 cell and transplanted to rho-0 (ρ0) cells. **A)** Assessment of ATP via cell titer glo with 48hr pretreatment of BrdU. **B & C)** Western blot of thymidine kinase and E1A in HEK293, ρ0, and post-mitochondrial transplant cells. Samples were prepared in laemmli buffer and loaded at 50 μg/lane onto a 10% acrylamide gel and blotted for thymidine kinase (TK1; 1:1000) and HEK293 marker, E1A (1:1000). GAPDH (1:10000) was used as a loading control.

Cell type		Pluriselect	Filter A	Filter B
MDA-MB-231	nDNA	-6.04	ND	-4.42
	mtDNA	0.34	0.52	1.19
MCF7	nDNA	-2.16	-7.05	-6.10
	mtDNA	2.71	1.05	3.77

Supplementary Table 1. ΔC_t of HEK293 whole cells and mitochondrial isolates for nDNA depletion and mtDNA enrichment, respectively.
